# Circulating Tumor Cell and Metabolites as Novel Biomarkers for Early-Stage Lung Cancer Diagnosis

**DOI:** 10.3389/fonc.2021.630672

**Published:** 2021-05-31

**Authors:** Lingling Wan, Qingyi Liu, Di Liang, Yongdong Guo, Guangjie Liu, Jinxia Ren, Yutong He, Baoen Shan

**Affiliations:** ^1^ Cancer Institute, The Fourth Hospital of Hebei Medical University/The Tumor Hospital of Hebei Province, Shijiazhuang, China; ^2^ Department of Thoracic Surgery, The Fourth Hospital of Hebei Medical University/The Tumor Hospital of Hebei Province, Shijiazhuang, China

**Keywords:** lung cancer, LC-MS untargeted metabolomics, circulating tumor cell, next-generation sequencing, diagnosis

## Abstract

**Background:**

Lung cancer is a malignant tumor that has the highest morbidity and mortality rate among all cancers. Early diagnosis of lung cancer is a key factor in reducing mortality and improving prognosis.

**Methods:**

In this study, we performed CTC next-generation sequencing (NGS) in early-stage lung cancer patients to identify lung cancer-related gene mutations. Meanwhile, a serum liquid chromatography-tandem mass spectrometry (LC-MS) untargeted metabolomics analysis was performed in the CTC-positive patients. To screen potential diagnostic markers for early lung cancer.

**Results:**

62.5% (30/48) of lung cancer patients had ≥1 CTC. By CTC NGS, we found that > 50% of patients had 4 commonly mutated genes, namely, *NOTCH1*, *IGF2*, *EGFR*, and *PTCH1*. 47.37% (9/19) patients had *ARIDH1* mutations. Additionally, 30 CTC-positive patients and 30 healthy volunteers were subjected to LC-MS untargeted metabolomics analysis. We found 100 different metabolites, and 10 different metabolites were identified through analysis, which may have potential clinical application value in the diagnosis of CTC-positive early-stage lung cancer (AUC >0.9).

**Conclusions:**

Our results indicate that NGS of CTC and metabolomics may provide new tumor markers for the early diagnosis of lung cancer.

## Introduction

According to statistics provided by the International Agency for Research on Cancer (IARC) of the World Health Organization, there were approximately 2.09 million new cases of lung cancer and approximately 1.76 million deaths from lung cancer worldwide in 2018 (1). Both the morbidity and mortality rank first among cancers, and the 5-year survival rate of lung cancer at stage IV is only 6% (2). The main reason for this high mortality is that early-stage lung cancer lacks typical clinical symptoms, and when it is diagnosed, it is often already in the advanced stage, or even has metastases. Therefore, early diagnosis of lung cancer is a key factor in reducing cancer-related death and improving prognosis. At present, the commonly used diagnostic strategies for lung cancer include imaging, such as computed tomography, sputum exfoliation cytology, serum tumor markers and bronchoscopy, which can easily result missed diagnoses and misdiagnosis. Therefore, it is urgently important to identify new biomarkers for the early diagnosis of lung cancer.

Circulating tumor cell (CTC) refers to tumor cells derived from tumor tissue that enter the peripheral blood circulation. Even in the early stage of tumor, tumor cells may flow into the circulatory system. Therefore, CTC is an important marker for liquid biopsy and can be used as a non-invasive and real-time monitoring tool for tumors to detect micrometastases (3, 4). A French study showed that CTC could be employed to detect early-stage lung cancer 4-5 years earlier than Low-Dose Computed Tomography (LDCT) in chronic obstructive pulmonary disease (COPD) patients (5). In the past 10 years, the clinical application of CTC has primarily focused on the number of CTCs (6, 7). In recent years, single-cell sequencing of CTC was performed by next-generation sequencing (NGS) technology, which provided a novel and precise tool for CTC research (8, 9). Our previous research (10) showed that LDCT combined with CTC may be a more effective method for early-stage lung cancer screening. Through NGS analysis of CTC, we identified three cancer-related genes *KIT*, *SMARCB1* and *TP53* in five CTC positive patients.

During the development of tumors, metabolites in the body change. More recently, researchers have turned to metabolomics to analyse specific metabolic markers for the early diagnosis of lung cancer (11). Untargeted metabolomics can qualitatively indentify differential metabolites between different groups, thereby screening potential disease markers. A clinical study on the early diagnosis of lung cancer, including 65 non-smoking female non-small cell lung cancer (NSCLC) patients, 6 benign lung tumors patients, and 65 healthy controls, determine that cysteine, serine and 1-monooleoylglycerol, as a biomarker panel, can be used to diagnose non-smoking female NSCLC (12). Musharraf et al. analysed the plasma of lung cancer patients, COPD patients, healthy smokers and healthy non-smokers using GC-MS technology and observed that the fatty acid and glucose levels of lung cancer patients were higher than those of patients in other groups (13). Research by Ding et al. suggests that glucose metabolism disorders may be closely related to lung cancer, as indicated by the presence of such metabolites as glycerol phosphate, lactic acid, acetyl-CoA, and 3-phosphoglycerate (14). However, the accuracy and reliability of the identification of metabolites are low, and no efficient diagnostic markers for lung cancer have been found.

Recent studies have verified that changes in cancer cell metabolites regulate the tumor microenvironment (15, 16), which is very important in the occurrence and development of tumors, because it has the role of connecting genotype and phenotype (17). As early as 1889, Paget proposed that the organ microenvironment (“soil”) can affect the planting, invasion, survival, and growth of specific tumor cells (“seeds”). In addition, CTC can survive in the peripheral blood through the immune escape mechanism and as latent tumor-initiating seeds that eventually break out to replace the host tissue (18). CTC exists in the early stages of cancer and can be detected earlier than imaging (19), and can be used for early diagnosis of cancer (20). Guo et al.’s study explored the role of CTC detection and metabolomics profiles in the prediction of early recurrence of lung cancer (21). In addition, CTC is a complete tumor cell, carrying all the information of the tumor, such as RNA, DNA, proteins, sugars, lipids and so on. The CTC of patients with gastric cancer or colorectal cancer has different single-cell metabolism profiles and is a potential biomarker for identifying specific cancer types (22). Certain lipid metabolites can be used to distinguish patients with lung cancer from patients with benign lung diseases (23). But so far, there is no relevant research to explore the correlation between CTC and metabolomics in cancer diagnosis. In this study, we performed CTC NGS and LC-MS untargeted metabolomics analyses in early-stage lung cancer patients, to identify lung cancer-related gene mutations and metabolites in CTC-positive patients, and analyze the value of early diagnosis of lung cancer.

## Materials and Methods

### Patients

A total of 48 pathologically diagnosed as lung cancer patients were enrolled in this study from the Fourth Hospital of Hebei Medical University from Dec 2018 to Jan 2019. All these patients and healthy controls were volunteers from LDCT lung cancer screening from HeBei Province (10, 24). None of the patients received preoperative radiotherapy or chemotherapy. Among the diagnosed lung cancer patients, 21 were male, and 27 were female. The age range was 38-75 years with an average age of 59.1 years. Postoperative pathology showed that all lung cancer patients were NSCLC, and 3 squamous cell carcinomas, 45 adenocarcinomas. According to the eighth edition of the TNM staging criteria, 41 cases were stage I, and 7 cases were stage IIa. And in this study, combined with clinical experience, we defined these I/IIa patients as early-stage lung cancer. All patients underwent CTC *in vivo* before surgery, and peripheral blood was taken for metabolomic detection. Meanwhile, 30 healthy controls were enrolled in the group, and their personal characteristics, such as gender, age, and smoking history, matched those of the lung cancer group, there was no significance. Approval was obtained from the Forth Hospital of Hebei Medical University ethics committee (Shijiazhuang, Hebei, China), and written informed consent was obtained from all the patients.

### Circulating Tumor Cell Analysis

#### CTC Capture and CTC PD-L1 Identifcation

The CellCollector^®^ (GILUPI GmbH, Potsdam, Germany) is a medical stainless-steel wire with a 2 cm functional area coated with EpCAM antibodies and hydrogel coatings. CellCollector^®^ was punctured into the peripheral blood of the cubital vein through a 20G indwelling needle and was held in the body for 30 minutes to capture tumor cells. After the collection of CTC was completed, the CellCollector^®^ with captured CTC is stained and identified according to the instructions of the staining kit, and a negative control (NK92 cells, Culture Collection of the Chinese Academy of Sciences, Shanghai, China) and positive control (SK-BR-3 cells, Culture Collection of the Chinese Academy of Sciences, Shanghai, China) were also provided. CD45 (Exbio, Clone Mem-28-Alexa647) antibody, cytokeratin 7/19/pan-CK antibody (Exbio Praha, Clone A53-B/A2-Alexa488) and PD-L1 (Clone PD-L1, Abcam) antibody staining analysis was performed, and nuclear staining was subsequently performed by Hoechst 33342 (Sigma) to identify tumor cells and analyse the expression of PD-L1 in CTC.

#### Whole Genomic Amplification and Next Generation Sequencing of CTC

After identifying CTCs by immunofluorescence staining, the CTC area under the microscope was located and sheared. A small portion of the CTCs contained in the sampling needle was collected into a PCR tube, and the MALBAC method was employed to perform whole-genome amplification. Qubit 3.0 and Nanodrop 2000 (Thermo Fisher) were used for quantitative analysis. The eligibility criteria were as follows: Qubit 3.0>10 ng/μl, ND 2000>40 ng/μl. Quantitative PCR (ABI7500) was employed to detect the coverage of some tumor driver gene fragments. Each amplified sample was tested for coverage of 8 different segments, and 5 or more coverages were qualified. If both the output and the coverage were observed to meet the requirements, the amplification product was determined to pass the inspection.

In total, 50 ng of genomic DNA (Nanodrop concentration as the standard) was used to construct sequencing libraries using the Ion Ampliseq Library Kit 2.0 (Thermo Fisher) and Ion Ampliseq Cancer Hotspot Panel v2 in keeping with the manufacturer’s instructions. Then, quality inspection and next-generation sequencing (NGS) were performed. NGS was performed with HiSeq X Ten (Illumina) following the manufacturer’s protocols using a paired-end 150-bp (PE150) sequencing strategy with a 127-gene panel.

### Metabolomics Analysis

#### Sample Preparation for Metabolomics

Thirty CTC-positive lung cancer patients were selected for fasting blood sampling. 5mL whole blood was collected into a sterile coagulation BD vacuum blood collection tube, immediately mix upside down for 5-8 times, place at 4°C for 30-120min, and centrifuge at 4°C 1300g for 10min, and transfer 0.2-1mL serum to 1.5mL EP tubes, saved at -80°C. Meanwhile, the 30 healthy volunteers were also took for fasting blood sampling, and serum preparation same as lung cancer group.

A 100-μL serum sample was collected, and the metabolite was extracted with 400 μL methanol:acetonitrile (1:1, v/v) solution. The mixture was vortexed for 30 seconds and sonicated on ice for 10 min, and this step was repeated 3 times. The sample was placed at -20°C for 30 min. After centrifugation at 13000 g at 4°C for 15 min, the supernatant was carefully transferred to a sample bottle for LC-MS/MS analysis.

#### Metabolite Detection

Metabolites were analyzed using the UPLC-Triple-TOF-MS-based platform (AB SCIEX, USA). The chromatographic separation of metabolites was performed using an ExionLCTMAD system (AB Sciex, USA) equipped with ACQUITY UPLC BEH C18 column (100 mm × 2.1 mm i.d., 1.7 μm; Waters, Milford, USA). Mobile phase A is water (containing 0.1% formic acid), mobile phase B is acetonitrile/isopropanol (1/1) (containing 0.1% formic acid); the flow rate is 0.40 mL/min, the injection volume is 20 μL, and the column temperature is 40 °C.

As part of the system adjustment and quality control process, a combined quality control sample (QC) was prepared by mixing all samples of equal volume. QC samples were injected at regular intervals (every 9 samples) to monitor the stability of the analysis. QC samples were treated and tested in the same way as analytical samples. It was preferable to represent the entire sample set, and to monitor the stability of the analysis.

#### Data Preprocessing and Annotation

After UPLC-TOF/MS analysis, the raw data were imported into Progenesis QI 2.3 (Waters Corporation, Milford, USA) for peak detection and comparison. The preprocessing result generated a data matrix consisting of retention time (RT), mass-to-charge ratio (m/z) values and peak intensity. At least 50% of the metabolic characteristics detected in all samples were retained. After filtering, half of the lowest metabolite value of a specific metabolite was estimated. In these specific samples, the metabolite level fell below the lower limit of quantification, and each metabolite characteristic was normalized by the sum (25). QC samples were used for data quality control, and delete the variables with the relative standard deviation (RSD) of quality control sample > 30% to obtain the final data matrix for subsequent analysis.

By matching with the database (http://www.hmdb.ca/, https://metlin.scripps.edu/), the metabolite list and data matrix were finally obtained. Differential metabolites were analyzed by Principal component analysis (PCA) and Orthogonal partial least squares discriminate analysis (OPLS-DA). The model validity was evaluated from model parameters R^2^ and Q^2^, which provide information for the interpretability and predictability, respectively, of the model and avoid the risk of over-fitting. Variable importance in the projection (VIP) were calculated in OPLS-DA model. P- values were estimated with paired Student’s t-test on Single dimensional statistical analysis. T-test combined with multivariate analysis OPLS-DA method was used to screen out the differential metabolites between groups (while meeting VIP >1, P-value <0.05).

Moreover, the classification information of differential metabolites was further obtained by comparing with HMDB 4.0 database (http://www.hmdb.ca/). And mapped into their biochemical pathways through metabolic enrichment and pathway analysis based on database search (KEGG, http://www.genome.jp/kegg/), so as to evaluate its influence on the biological metabolism process.

### Statistical Analysis

The data were analyzed by SPSS 22.0 software. The measurement data followed the normal distribution using the mean ± standard deviation, those not following the normal distribution used the median (quartile), and the counting data used frequency or rate. A t-test was used for the comparison of measurement data in a normal distribution, and a rank sum test was used for the comparison of measurement data in a non-normal distribution. The chi-square test was used to compare counting data. ROC curves were used to analyze the diagnostic effect of different indexes on lung cancer. P-values < 0.05 were considered to be significant.

## Results

### CellCollector^®^ *In Vivo* CTC Detection

**Figure 1:**
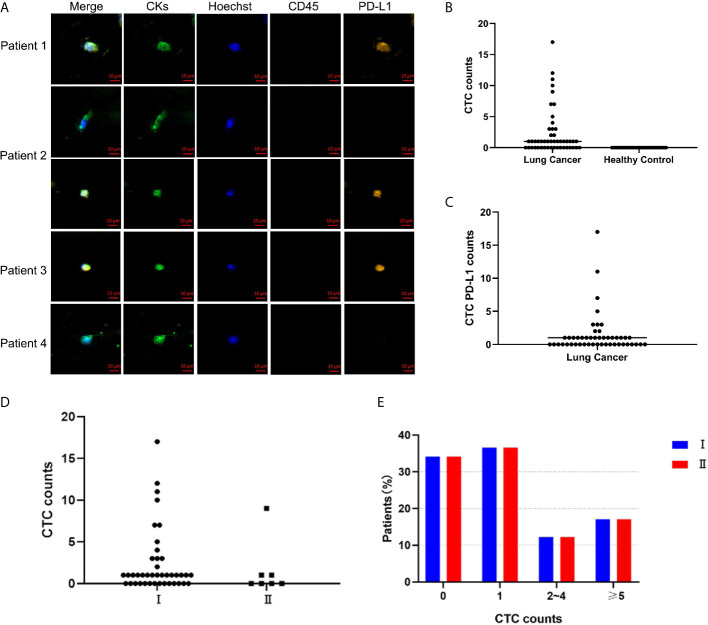
CellCollector captures representative CTC graphs and CTC and CTC PD-L1 detection. **(A)** CellCollector captures CTC identification charts. Patient 1 captures 7 CTCs, patient 2 captures 3 CTCs, patients 3 and 4 capture 1 CTC. **(B)** Detection rate and number of CTC in lung cancer patients and healthy controls. **(C)** Detection rate of PD-L1+ CTC in patients with lung cancer. **(D)** Detection rate and number of CTC in stage I and stage II lung cancer patients. **(E)** Distribution of detected CTCs in stage I and stage II patients.

A total of 30 of 48 patients had ≥1 CTC detected with the CellCollector^®^ *in vivo* strategy, and the detection rate was 62.5% (range, 0-17, median, 1). **Figure 1A** is a representative diagram of CTC capture. No CTC was detected in healthy controls (**Figure 1B**). The detection rates of CTC were 64% (16/25) and 60.86% (14/23) in the <60-year-old patients and ≥ 60-year-old patients, respectively. The detection rates among male and female patients were 66.67% (14/21) and 59.26% (16/27), respectively. Regardless of whether the patients smoked, the detection rate was equivalent, 63.33% and 61.11%, respectively. In addition, we also found that the detection rate of stage I lung cancer patients was 65.85% (27/41), and the detection rate of stage II patients was 42.86% (3/7) (**Figure 1D**). **Figure 1E** is the distribution of the number of CTCs in I/II patients, there was no correlation between the number of CTC and stage (**Figure 1E**). There was no correlation between CTC and clinical characteristics (**Table 1**). Meanwhile, PD-L1 protein was detected and 52.08% (25/48) of patients had PD-L1 expression on CTC (**Figure 1C**).

**Table 1:** Clinical characteristics of patients.

Patients characteristic		CTC		p-value	PD-L1+ CTC		p-value
		negative	positive		negative	positive	
Age							
<60 years		9(36.00%)	16(64.00%)	0.823	13(52.00%)	12(48.00%)	0.555
≥60years		9(39.13%)	14(60.87%)		10(43.48%)	13(56.52%)	
Gender							
Male		7(33.33%)	14(66.67%)	0.599	11(52.38%)	10(47.62%)	0.585
Female		11(40.74%)	16(59.26%)		12(44.44%)	15(55.56%)	
Smoking							
No		11(36.67%)	19(63.33%)	0.878	13(43.33%)	17(56.67%)	0.412
Yes		7(38.89%)	11(61.11%)		10(55.56%)	8(44.44%)	
Stage							
I		14(34.15%)	27(65.85%)	0.460	20(48.78%)	21(51.22%)	1.000
II		4(57.14%)	3(42.86%)		3(42.86%)	4(57.14%)	
Tumor size							
<1.5cm		11(45.83%)	13(54.17%)	0.233	12(50.00%)	12(50.00%)	0.773
≥1.5cm		7(29.17%)	17(70.83%)		11(45.83%)	13(54.17%)	

CTC, circulating tumor cell.

### Next Generation Sequencing of CTC

**Figure 2:**
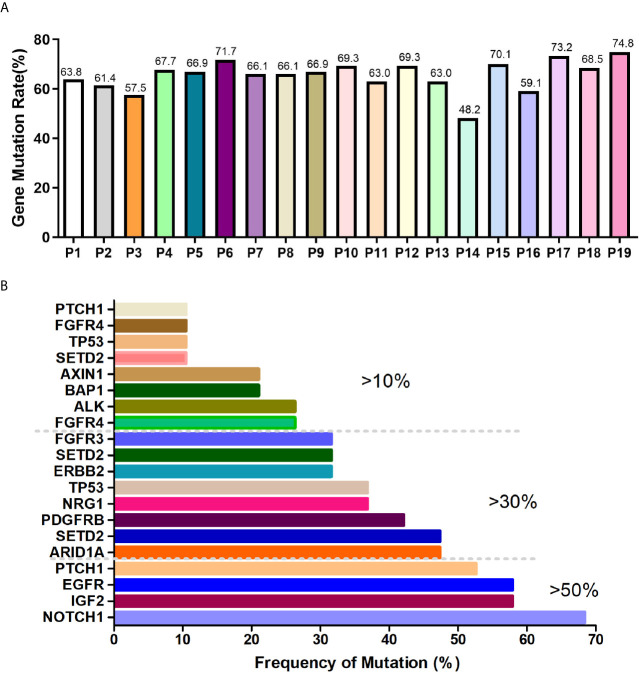
CTC NGS analysis of cancer-related gene mutations in lung cancer patients. **(A)** Proportion of mutated genes in 19 lung cancer patients. **(B)** >10%, >30%, >50% of patients with mutated genes.

Preamplification and quality inspection were performed on 30 patients with CTC. 19 randomly selected samples were qualified for quality inspection. NGS was performed to analyze 127 cancer-related mutated genes (**Figure S1**). The gene mutation rate of 19 patients was 48.2% to 74.8%, among which 4 patients had a gene mutation rate of >70%, 12 patients had a gene mutation rate between 60% and 70%, 2 patients had a gene mutation rate between 50% and 60%, and 1 patient had a gene mutation rate below 50% (**Figure 2A**). To explore whether the lung cancer-related mutant genes could be screened early by CTC gene detection, we conducted a common mutation analysis on 19 CTC sequencing samples. According to the proportion of patients with common genetic mutations, we are divided into 3 groups, which are >10%, >30% and >50% (**Figure 2B** and **Table 2**). Among these groups, >50% of patients had 4 common mutation genes, namely, *NOTCH1*, *IGF2*, *EGFR* and *PTCH1*. 47.37% (9/19) of patients had mutated *ARID1A*. Gene information and mutation sites are shown in **Table 2**. Based on analysis of the COSMIC database, *NOTCH1* and *ARID1A* are reported to be associated with lung cancer.

**Table 2:** Mutation gene information in >10%, >30% and >50% patients.

Gene	Mutation site	Mutation frequency (%)	Gene annotation
NOTCH1	NM_017617:exon34:c.7244_7246del:p.2415_2416del	68.42	It belongs to the NOTCH family, and abnormal signals are associated with the occurrence of tumors.
IGF2	NM_001127598:exon5:c.686delC:p.P229fs	57.89	Insulin-like growth factor 2
PTCH1	NM_000264:exon22:c.3606delC:p.P1202fs	52.63	The negative regulatory gene of hedgehog signaling pathway. Hedgehog signaling pathway is associated with tumorigenesis and development
EGFR	NM_005228:exon9:c.1078delA:p.K360fs	57.89	A member of the epidermal growth factor receptor family, associated with tumor cell proliferation, angiogenesis, tumor invasion, and metastasis.
ARID1A	NM_006015:exon20:c.5542delG	47.37	Potential tumor suppressor gene
TP53	NM_000546:exon2:c.58delT:p.S20fs	36.84	Tumor suppressor gene
FGFR3	NM_001163213:exon18:c.2336delC:p.T779fs	31.58	Recombinant human fibroblast growth factor receptor-3, somatic FGFR3 mutations have been reported to be more common in superficial papillary bladder tumors.
PDGFRB	NM_002609:exon19:c.2594delT:p.L865fs	42.11	Human platelets derive growth factor receptors
ERBB2	NM_001005862:exon20:c.1911delG:p.L637fs	31.58	A member of the epidermal growth factor receptor family, associated with tumor metastasis and prognosis
SETD2	NM_014159:exon3:c.164delT:p.L55fs	47.37	Potential tumor suppressor gene, related to poor prognosis of tumor
SETD2	NM_014159:exon3:c.101delA:p.N34fs	31.58	Potential tumor suppressor gene, related to poor prognosis of tumor
NRG1	NM_001160004:exon10:c.934delA:p.K312fs	36.84	Epidermal growth factor receptor (EGFR) is a member of the epidermal growth factor receptor family
AXIN1	NM_003502:exon7:c.1922delA:p.K641fs	21.05	It is associated with ontogenesis, cell proliferation and carcinogenesis
TP53	NM_000546:exon4:c.98C>T:p.S33F	10.53	Tumor suppressor gene
ALK	NM_004304:exon7:c.1435delT:p.Y479fs	26.32	Oncogenic driver gene
SETD2	NM_014159:exon3:c.4319delC:p.P1440fs	10.53	Potential tumor suppressor gene, related to poor prognosis of tumor
BAP1	NM_004656:exon13:c.1464delC:p.P488fs	21.05	Closely related to tumor development
FGFR4	NM_022963:exon4:c.453delC:p.H151fs	26.32	Fibroblast growth factor, related to angiogenesis, involved in tumor relapse resistance
FGFR4	NM_022963:exon6:c.734delC:p.S245fs	10.53	Fibroblast growth factor, related to angiogenesis, involved in tumor relapse resistance
PTCH1	NM_001083604:exon23:c.3734G>A:p.G1245E	10.53	The negative regulatory gene of hedgehog signaling pathway. Hedgehog signaling pathway is associated with tumorigenesis and development

### Multivariate Analysis for Discriminating Metabolites Between Lung Cancer Patients and Control Individuals

To determine whether there are specific differential metabolites in the early-stage of lung cancer, this study used LC-MS to perform metabolomics analysis on serum samples of lung cancer patients and control individuals. Typical total ion current (TIC) chromatograms of metabolic profiles analyzed using UPLC-Triple TOF-MS/MS in the positive mode or negative mode are shown in **Figures S2A–D**. The peaks are abundant and uniform and no obvious differences between the lung cancer group and the healthy groups were detected. In order to evaluate the stability of the analysis system during the on-boarding process, QC samples are necessary, which are a mixture of all samples in equal volumes. During data analysis, the stability of the instrument during the entire analysis process can be investigated through the repeatability of QC samples, Meanwhile, it can also be used to find the variable with great variation in the analysis system to ensure the reliability of the results. PCA suggested that the QC samples were clustered closely, verifying the good repeatability of the UPLC-Triple TOF-MS/MS method (**Figures S2E, F**). PCA score plots and heat map show the distribution of metabolites in lung cancer group and healthy control group in the positive/negative ion mode. The metabolite profiles showed that the early lung cancer group and the healthy control group could be significantly separated, and the lung cancer group samples had high similarity (**Figure S2**).

**Figure 3:**
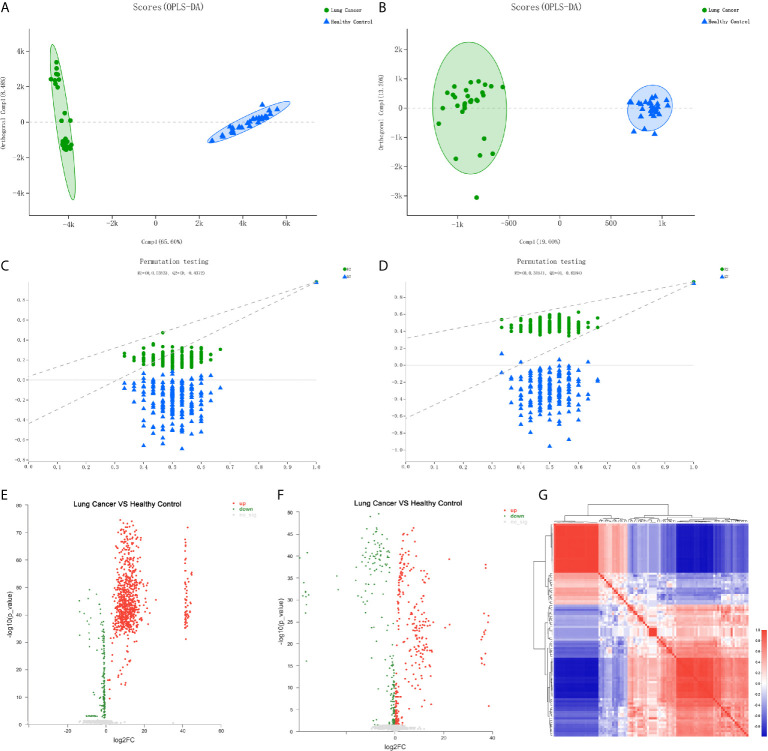
Differential metabolite analysis of serum from the lung cancer group and the healthy control group. **(A)** OPLS-DA score of the lung cancer group and the healthy control group in the negative ion mode. **(B)** OPLS-DA score of the lung cancer group and the healthy control group in the positive ion mode. **(C)** OPLS-DA differential metabolite serum anion permutation testing. R^2^ = (0, 0.0353), Q^2^ = (0, -0.4372). **(D)** OPLS-DA differential metabolite serum cation permutation testing. R^2^ = (0, 0.3151), Q^2^ = (0, -0.6284). **(E)** Volcano plot of serum anion metabolites in the control group and lung cancer group. **(F)** Volcano plot of differential metabolites of serum cations in the healthy controls and lung cancer group. **(G)** Metabolite heat map of serum differences between healthy controls and lung cancer groups.

To further identify the differential metabolic characteristics of the early-stage lung cancer group, the differential metabolites between groups were screened by t- test combined with multivariate OPLS-DA analysis, which showed some differences in the metabolites between the lung cancer group and healthy groups under both ionization modes (**Figures 3A, B**). The quality of OPLS-DA was evaluated by R^2^Y and Q^2^ values, which were calculated as 0.035 and -0.437 in the negative ion mode, 0.031 and -0.624 in the positive mode (**Figures 3C, D**). Combined with VIP >1, P <0.05 and FC >1 or FC <1, the volcano chart shows that there were up-regulated or down-regulated metabolites among the 100 differential metabolites (**Figures 3E, F**). The most significant upregulation of differential metabolites in the early lung cancer group involved the lipid material, especially sphingolipids (such as trihexylceramide) and glycerophospholipids (such as cardiolipin), which are components of the cell membrane, and the differential metabolites are most significantly downregulated. Cyclic guanosine phosphate and guanosine 1-phosphate play important roles in purine nucleotide metabolism. In short, it is believed that these 100 differential metabolites are closely related to early-stage lung cancer (**Figure 3G**).

### Metabolic Pathway Analysis of the Differentially Regulated Metabolites

**Figure 4:**
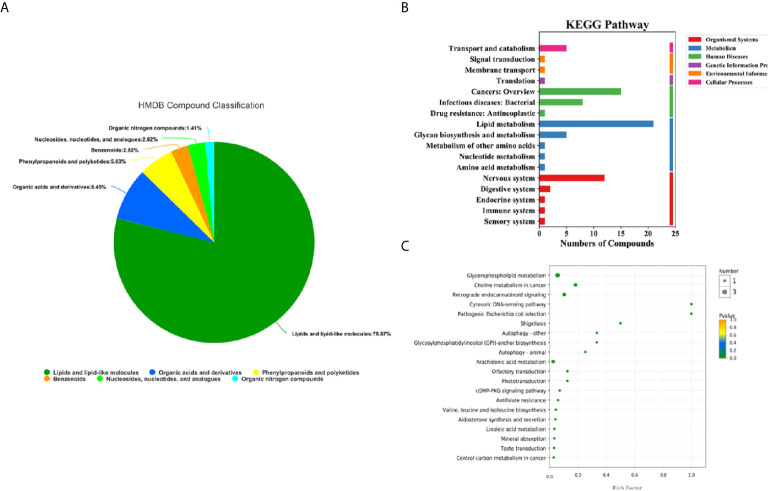
HMDB compound classification and KEGG pathway analysis of serum metabolites in the lung cancer group and healthy control group. **(A)** HMDB compound classification of serum metabolites between the lung cancer group and the healthy control group. **(B)** Serum KEGG pathways between the lung cancer group and the healthy control group. **(C)** KEGG enrichment pathways for serum metabolites in the lung cancer group and the healthy control group.

100 differential metabolites screened were classified into compounds by the Human Metabolome Database (HMDB), and the KEGG (Kyoto Encyclopedia of Genes and Genomes) database was used for pathway annotation and enrichment analysis. There were 6 types of differential metabolites that we screened (**Figure 4A**), most of which were lipids and their analogues, accounting for 78.87%, followed by organic acids and their derivatives, accounting for 8.45%, and phenylpropyl esters, accounting for 5.63%. The KEGG pathway analysis was divided into six categories (**Figure 4B**). The three most significant pathways for enrichment through KEGG are choline metabolism in cancer, glycerophospholipid metabolism, and the retrograde endocannabinoid signaling pathway. Enrichment analysis of the KEGG pathway revealed that the highest rate of differential metabolite enrichment was the cytosolic DNA-sensing pathway and the pathogenic *Escherichia coli* infection pathway (**Figure 4C**).

Previous studies have found that the occurrence and development of tumors are closely related to glucose and lipid metabolism (26, 27). In this study, according to KEGG pathway analysis, there were more differential metabolites enriched in the glucose and lipid metabolism pathways and tumor metabolism pathways between the two groups (**Figure 4B**). Among these metabolites, there were 21 differential metabolites involved in lipid metabolism pathways (**Table S1**), 5 differential metabolites involved in polysaccharide synthesis and metabolic pathways (**Table S2**), and 15 differential metabolites involved in tumor metabolic pathways (**Table S3**). We are currently conducting a targeted metabolomics study in another large cohort using UPLC -MS/MS. One of the results of a small sample study (5 lung cancer test groups and 5 healthy control groups) showed that phosphatidylethanolamine is up-regulated, which is similar to the results of this study (**Figure S3**).

### Evaluation of the Metabolic Index in the Diagnosis of Lung Cancer

**Figure 5:**
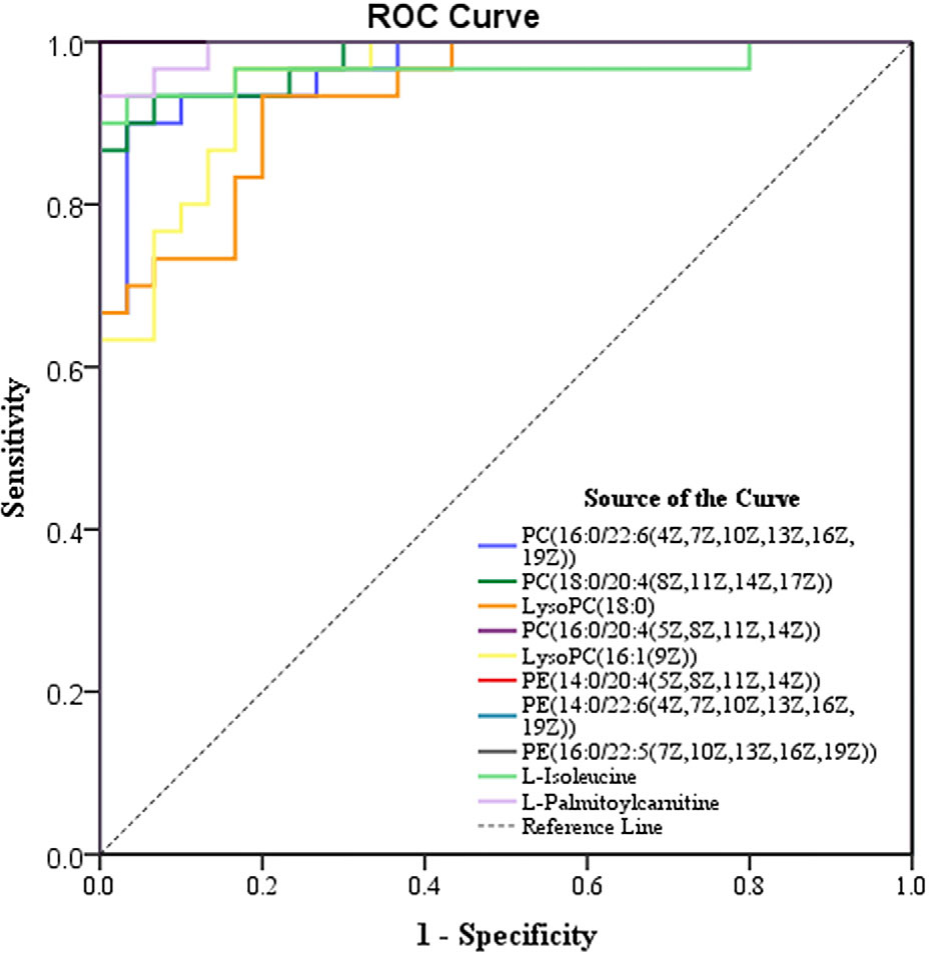
ROC curve of nine metabolites in the diagnosis of lung cancer.

To effectively screen potential biomarkers in the early-stage lung cancer group, we further compared the differential metabolites in lipid metabolic pathways, polysaccharide synthesis and metabolism pathways, and tumor-related pathways between the early-stage lung cancer group and the healthy control group using receiver operating characteristic (ROC) curve and area under the curve (AUC) (**Figure S4**). It was found that AUC > 0.9 contained 9 differential metabolites related to lipid pathways, 3 differential metabolites related to polysaccharide synthesis and metabolism, and 5 differential metabolites related to tumor pathways. These metabolites were primarily glycerophospholipids, which are integrated with 3 types of upregulated differences and 7 types of downregulated difference, namely, PE (14:0/22:6(4Z,7Z,10Z,13Z,16Z,19Z)), PE (16:0/22:5(7Z,10Z,13Z,16Z, 19Z)), PE (14:0/20:4(5Z,8Z,11Z,14Z)), PC (18:0/20:4(8Z,11Z,14Z,17Z)), PC (16:0/22:6(4Z,7Z,10Z,13Z,16Z,19Z)), PC (16:0/20:4(5Z,8Z,11Z,14Z)), LysoPC (16:1(9Z)/0:0), L-isoleucine, LysoPC (18:0), L-palmitoylcarnitine (**Figure 5** and **Table 3**).

**Table 3:** The diagnostic efficacy of various metabolites in lung cancer.

Metabolite	AUC	95%CI	p-value	Sensitivity	Specificity
PC[16:0/22:6(4Z,7Z,10Z,13Z,16Z,19Z)]	0.968	0.930~1.000	<0.001	90.00%	96.67%
PC[18:0/20:4(8Z,11Z,14Z,17Z)]	0.979	0.950~1.000	<0.001	93.33%	93.33%
LysoPC(18:0)	0.933	0.876~0.991	<0.001	93.33%	80.00%
PC[16:0/20:4(5Z,8Z,11Z,14Z)]	1.000	1.000~1.000	<0.001	100.00%	100.00%
LysoPC[16:1(9Z)]	0.951	0.904~0.998	<0.001	96.67%	83.88%
PE[14:0/20:4(5Z,8Z,11Z,14Z)]	1.000	1.000~1.000	<0.001	100.00%	100.00%
PE[14:0/22:6(4Z,7Z,10Z,13Z,16Z,19Z)]	1.000	1.000~1.000	<0.001	100.00%	100.00%
PE[16:0/22:5(7Z,10Z,13Z,16Z,19Z)]	1.000	1.000~1.000	<0.001	100.00%	100.00%
L-Isoleucine	0.967	0.914~1.000	<0.001	93.33%	96.67%
L-Palmitoylcarnitine	0.993	0.9817~1	<0.001	93.33%	100.0%

## Discussion

In this study, CTC counting, CTC next-generation sequencing, and LC-MS untargeted metabolomics were combined to characterize the potential gene mutation and energy metabolism disturbance characteristics of lung cancer, to provide a better detection method for the early screening and diagnosis of lung cancer patients. We used CellCollector^®^ *in vivo* CTC capture technology to detect CTC in early lung cancer patients. The CTC detection rate was 62.5%. Compared with *in vitro* technology, it has a higher detection rate, which is consistent with previous research results (24, 28). Because the content of CTC in the peripheral blood of tumor patients is very low, the number is even rarer in precancerous lesions or early tumors. In our study, the median CTC was 1 in stage I/II NSCLC patients and 0 in healthy volunteers. CTC detection can be used for the early diagnosis of lung cancer, which is also consistent with previous studies (20). Unfortunately, our study cannot find out whether the number of CTCs is correlated with different stages of lung cancer, which may be related to the small sample size, of which only 7 cases were in stage II. This is also what we need to increase the sample size and patients with different stages of lung cancer to further explore in our subsequent studies.

The high CTC detection rate provides convenience for CTC molecular typing and CTC next-generation sequencing. Through CTC NGS, this study found that more than 50% of early lung cancer patients have 4 common mutated genes, namely *NOTCH1*, *IGF2*, *EGFR* and *PTCH1*. Also, 47.37% of patients have *ARID1A* mutations. *EGFR* has the highest mutation rate in NSCLC (29). *NOTCH1*, a member of the PCG gene family, was first discovered in mouse B-cell lymphoma and is regarded as a co-oncogene of C-MYC, closely related to cell proliferation, differentiation and apoptosis (30). Studies have shown that the stimulation of the Notch signaling pathway by high NOTCH1 expression can induce BM-1 to mediate the production of related intracellular signals to accelerate the transmission efficiency of lung cancer, thereby inducing the occurrence, development, metastasis and invasion of lung cancer (31). In our study, 68.42% of patients had a mutation in NOTCH1. Huang et al. (32) found that homozygous *ARID1A* was deleted at the 5’ end of the lung adenocarcinoma cell line, strongly suggesting that *ARID1A* is a tumor suppressor gene. Imielinska et al. reported exons and genome sequences of 183 cases of lung adenocarcinoma, and the results showed that mutations of the *ARID1A* gene existed in lung adenocarcinoma cells (33). ARID1A is a member of the SWI/SNF family and can regulate the expression of specific genes by changing the structure of chromatin. It is currently considered as a potential tumor suppressor gene, which plays an important role in inhibiting cell proliferation, inhibiting cell metastasis, and promoting cell differentiation and apoptosis. Frequent mutations in a variety of human malignancies indicate that *ARID1A* plays an important role in the occurrence and development of human malignancies. Among them, the expression of ARID1A in NSCLC was significantly lower than that of normal bronchial epithelial cells. In addition, *ARID1A* mutations are related to the activation of PI3K/AKT signaling pathway. However, the mutations and prognostic significance of ARID1A gene in different studies are different. There are still some problems to be solved, such as the target genes regulated by ARID1A and the specific mechanisms in tumor suppression, whether other signaling pathways can exert tumor suppression effects, and the prognostic value of ARID1A and patients. This requires comprehensive and in-depth research (34). Our study is the first to find *IGF2* and *RTCH1* mutations in peripheral blood CTC NGS of early lung cancer. Whether these mutations can be combined with *NOTCH1*, *EGFR* and *ARID1A* mutations as tumor markers in the diagnosis of early lung cancer merits further investigation.

Studies have found that CTC already exists in the early stages of cancer (35), and disturbances of metabolism are produced in the body (36), including disorders of glucose and lipid metabolism (13, 27, 37, 38), and the homeostasis of the microenvironment of the body is disrupted. Through metabolomics analysis, we found 100 different metabolites, which mainly occurred in lipid metabolism, polysaccharide synthesis and metabolism, amino acid metabolism and other pathways and were dominated by lipid metabolism, being especially enriched in choline metabolism and glycerophospholipid metabolic pathways. Chen et al. found that abnormal sphingolipid metabolism is the most important metabolic change in lung cancer patients (39). A study on lung adenocarcinoma in female non-smokers found that abnormal lipid metabolism may play role in the development of lung cancer (12). High-lipid molecules, including phospholipids (e.g., glycerophospholipids and sphingomyelin), and cholesterol are the main component of cell membranes and participate in cell signaling and cell proliferation. Lipid metabolism changes cause abnormal cell signals and lead to tumor formation (40, 41).

Tumor growth requires the uptake of a large amount of energy in the blood. The body ensures the normal energy metabolism of other organs by increasing the “raw materials” in the aerobic oxidation pathway, resulting in increased glucose metabolism and decreased fat metabolism (42). The purpose of this study was to discover a combination of serum metabolite biomarkers for the early detection of non-small cell lung cancer. In our study, the most obvious differences in the screened metabolites can be divided into four categories, namely phosphatidylethanolamine (PE), phosphatidylcholine (PC), lysophosphatidylcholine (LysoPC), L-isoleucine and L-palmitoylcarnitine. A large number of metabolomic studies have been undertaken to identify robust biomarkers for lung cancer diagnosis using plasma, serum, or urine. However, we found remarkably few metabolomic studies that specifically attempted to detect early-stage lung cancer.

The concentration of LysoPC was reduced in stage I/II NSCLC, which is similar to previous research (43). Another targeted metabolomics study found and verified that β-hydroxybutyric acid, LysoPC 20:3, PC ae C40:6 (a kind of phosphatidylcholines), citric acid, and fumaric acid differed significantly between healthy controls and stage I/II NSCLC. Robust predictive models with AUC >0.9 were developed and validated using these metabolites and other, easily measured clinical data for detecting different stages of NSCLC (44). It has been observed and reported that in mouse and human models, the plasma concentration of total LysoPC is usually inversely related to the risk of various types of cancer (45–47). In our study, we found that another member of the phosphatidylcholine family, PC(16:0/22:6(4Z,7Z,10Z,13Z,16Z,19Z)), PC(18:0/20:4(8Z,11Z,14Z,17Z)) and PC(16:0/20:4(5Z,8Z,11Z,14Z)), also appears to play a role in both stage I and stage II NSCLC. A study reported that PC levels were dysregulated in early-stage NSCLC patients (48). Decreased lipid membrane unsaturation levels were observed to protect tumor cells from free radicals or chemotherapeutics and promote invasion and infiltration (49). Clearly, more detailed lipidomic studies need to be conducted to investigate the biological significance of these PC alterations.

Lysophosphatidylethanolamine (LPE) is a group of lipids that has been recently shown to be related to breast cancer (50). In addition, PE (16:0/18:1) is associated with the stage and prognosis of pancreatic cancer and may be a potential diagnostic marker (51). Yang et al. found 25 different lipid metabolites, including PE, between malignant pleural effusion (MPE) and benign pleural effusion (NPE), indicating that lipid metabolites may be used to partition MBE and BPE (52). In our study, PE (14:0/22:6(4Z,7Z,10Z,13Z,16Z,19Z)), PE (16:0/22:5(7Z,10Z,13Z,16Z, 19Z)), and PE (14:0/20:4(5Z,8Z,11Z,14Z)) were upregulated and effectively distinguished the control group, with specificity and sensitivity close to 100% being observed. In addition, we found that the level of amino acids (L-isoleucine) was significantly increased in the lung cancer model group compared with that of the control group, indicating disorder in amino acid metabolism in the cancer model group. Maeda et al. reported 6 significantly different amino acid metabolites, with AUCs of 0.817 and 0.801 (on their validation sets), for diagnosing stage I and stage II lung cancer (53). One Study showed that L-palmitoylcarnitine is significantly reduced in advanced lung cancer patients (54). In another study, the level of palmitoylcarnitine was lower in the hepatocellular carcinoma group than in the cancer-free control group, and blood acylcarnitine levels may be influenced by hepatic fatty acid metabolism, in other words, decreased acylcarnitine levels may reflect the decreased production of acyl groups in the liver or other tissues. Indeed, palmitoylcarnitine and palmitic acid are associated with fatty acid metabolism, and this group displayed an impact factor of 0.030 based on metabolic pathway analysis (55). The decrease in L-palmitoylcarnitine in our study may also be related to a disorder of lipid metabolism in patients with lung cancer.

This study is based on CTC NGS and metabolomics analysis to conduct a comprehensive assessment of early lung cancer, in order to find new biomarkers and improve the diagnosis rate of early lung cancer. Previous studies have reported that the CTC single-cell metabolism profile may provide direct functional insights into the tumor cell metabolism of patients, and promote a more direct understanding of the relationship between cancer cell genotype and metabonomic phenotype (22). Whether CTC gene mutations and metabolomics can be used for the early diagnosis of lung cancer, we will re-enroll patients and conduct targeted verification, and finally analyze the scoring algorithm to select the best markers suitable for early diagnosis. However, there are still some shortcomings to this research. First, due to the small sample size in this study, a large sample study is needed to further verify the reliability of our research results. Second, the differential metabolites screened by untargeted metabolomics were only detected by one cohort, and not further verified by targeted metabolomics. Additional study is necessary to more fully explore and validate the metabolic changes detected in this study in NSCLC patients. And we are cognizant of these aspects and are accordingly in the process of a targeted metabolomics study in another large cohort using UPLC -MS/MS. In the end, we only performed CTC gene mutation detection but did not sequence the tissue. Whether there is a relationship between the mutations carried by CTCs and the changes in metabolic substances, which affect the microenvironment of patients, and whether there is a connection with the occurrence and development of lung cancer warrants further study.

## Conclusions

In our study, there was a higher CTC detection rate, with 62.5% in I/II NSCLC, and 4 high frequency mutation genes, namely, NOTCH1, IGF2, EGFR and PTCH1. Meanwhile, we found that 10 different metabolites may have potential clinical application value for the diagnosis of CTC-positive early-stage lung cancer (AUC > 0.9). Later, a larger cohort of patients will be required for verification, which may help determine whether these markers can be used for the early diagnosis of lung cancer.

## Data Availability Statement

The original contributions presented in the study are publicly available. This data can be found here: https://figshare.com/s/5fe7f423c20532952ddd DOI: 10.6084/m9.figshare.14680037.

## Ethics Statement

The studies involving human participants were reviewed and approved by Approval was obtained from the Forth Hospital of Hebei Medical University ethics committee (Shijiazhuang, Hebei, China). The patients/participants provided their written informed consent to participate in this study.

## Author Contributions

Conceptualization, BS. Data curation, DL and YG. Formal analysis, LW. Funding acquisition, YH. Investigation, YG and JR. Methodology, QL. Project administration, QL. Resources, GL. Supervision, YH. Validation, LW. Visualization, DL. Writing – original draft, LW. Writing – review & editing, YH and BS. All authors contributed to the article and approved the submitted version.

## Funding

This work was supported by the Financial Department of Hebei Province [No. [2016] 361006 and No. 2017043367-2].

## Conflict of Interest

The authors declare that the research was conducted in the absence of any commercial or financial relationships that could be construed as a potential conflict of interest.

## Abbreviations

CTC, circulating tumor cell; NGS, next-generation sequencing; LC-MS, liquid chromatography-tandem mass spectrometry; IARC, International Agency for Research on Cancer; LDCT, Low-Dose Computed Tomography; COPD, chronic obstructive pulmonary disease; NSCLC, non-small cell lung cancer; QC, quality control; RT, retention time; RSD, relative standard deviation; FC, fold change; HMDB, Human Metabolome Database; KEGG, Kyoto Encyclopedia of Genes and Genomes; ROC, receiver operating characteristic; AUC, area under the curve. PE, Phosphatidylethanolamine; PC, Phosphatidylcholine; LysoPC, lysophosphatidylcholine.
