# Supplementary material for: Circulating Tumor Cell and Metabolites as Novel Biomarkers for Early-Stage Lung Cancer Diagnosis
Source: Front Oncol. 2021 May 31;11:630672. doi: 10.3389/fonc.2021.630672 (PMC8202280; doi:10.3389/fonc.2021.630672)
Supplement: Supplementary file 1 [file DataSheet_1.doc]

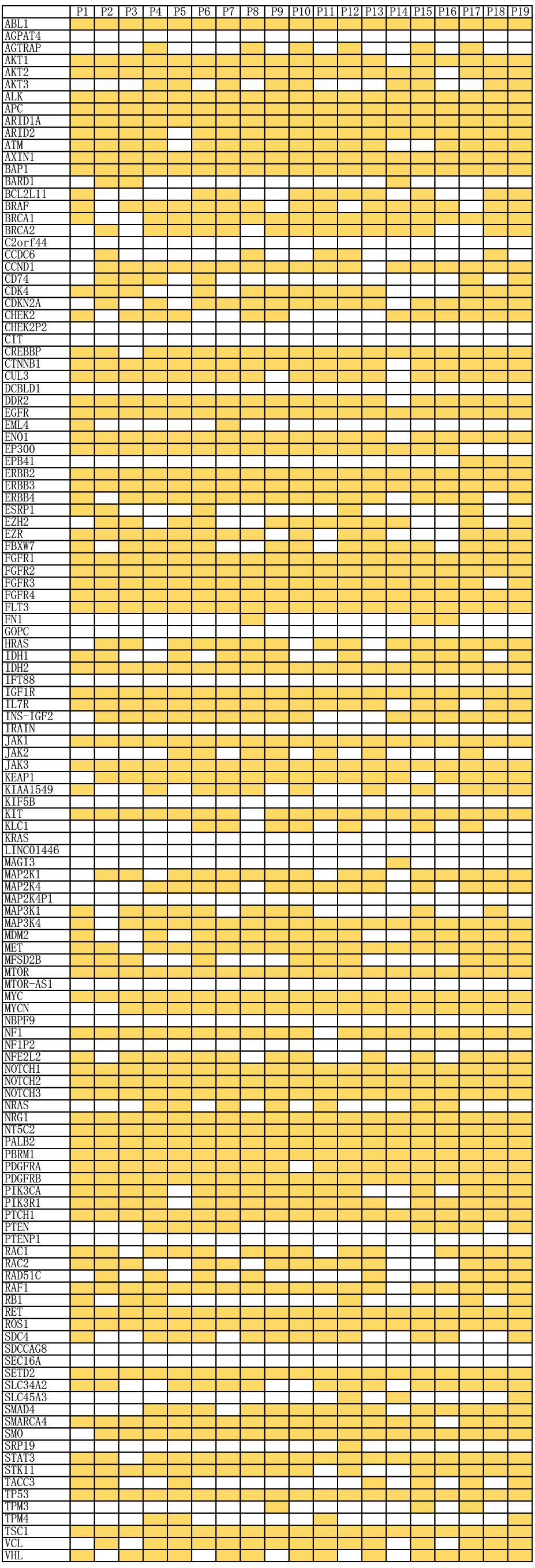


**Figure S1** Mutations in 127 genes in 19 CTC-positive patients. The yellow colour indicates that the gene is abnormal.


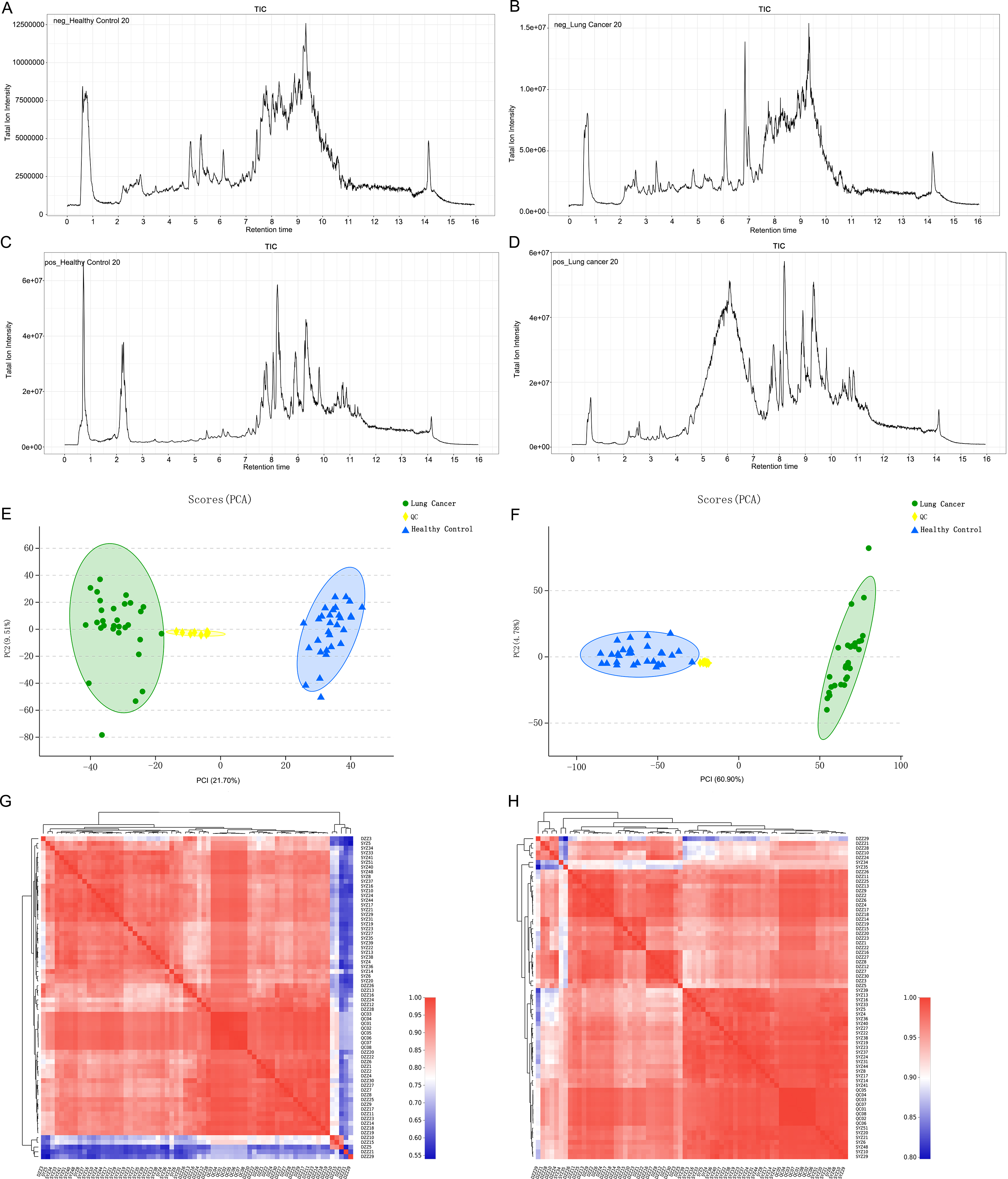


**Figure S2** Principal component analysis (PCA) score plots and heat map show the distribution of metabolites in lung cancer group and healthy control group. (A) Serum anion TIC analysis chart of healthy controls. (B) Serum anion TIC analysis chart of lung cancer patients. (C) Serum cationic TIC analysis chart of healthy controls. (D) Serum cationic TIC analysis chart of lung cancer patients. (E) PCA score plots in the negative ion mode between QC samples, healthy controls and lung cancer patients. (F) PCA score plots in the positive ion mode between QC samples, healthy controls and lung cancer patients. (G) Heat map of the healthy controls and lung cancer patients in the negative ion mode. (H) Heat map of the healthy controls and lung cancer patients in the positive ion mode.


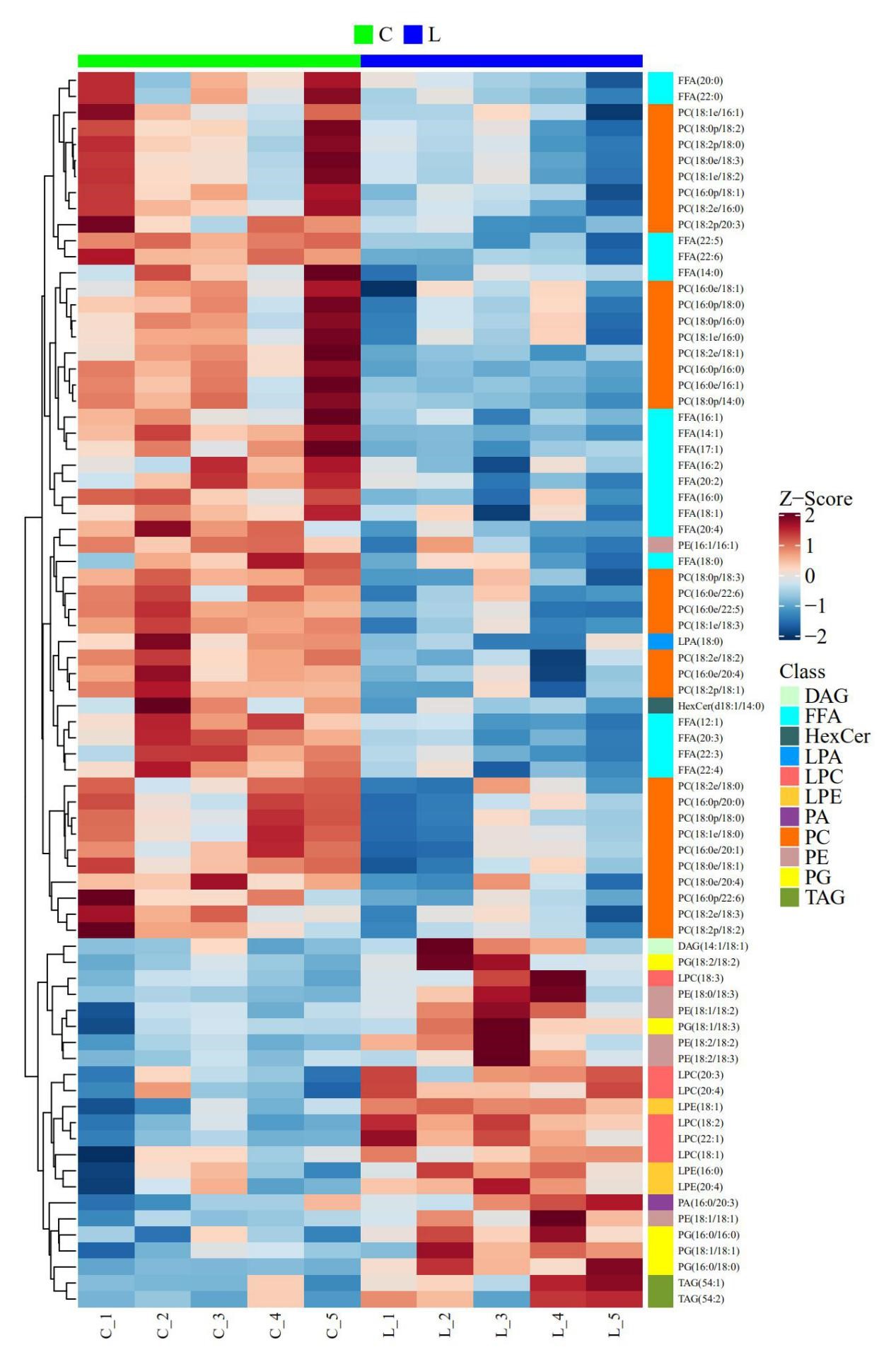
 **Figure S3** Heat map of differential metabolites between the lung cancer group and the control group in the validation group under UPLC -MS/MS. C，healthy control group. L, lung cancer group.


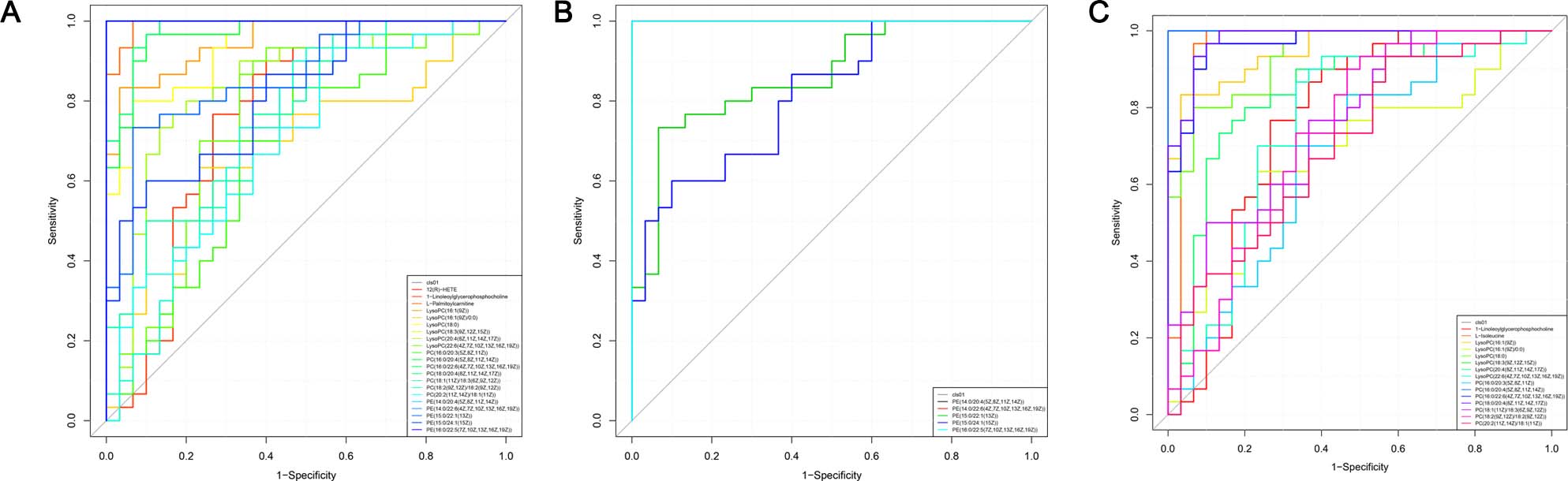


**Figure S4** ROC curve was used to analyse potential tumor markers in serum. (A) ROC curves of 21 differential metabolites in lipid metabolism pathways. (B) ROC curves of 5 different metabolites in the synthesis and metabolism of polysaccharides. (C) ROC curves of 15 differential metabolites in tumor-related pathways.

Table S1 Differential metabolite KEGG pathway - 21 metabolites in lipid metabolism

| ID | Metabolite | M/Z | Formula | Retention time |
| --- | --- | --- | --- | --- |
| pos_135 | LysoPC(22:6(4Z,7Z,10Z,13Z,16Z,19Z)) | 568.3383944 | C30H50NO7P | 7.589166667 |
| pos_137 | LysoPC(20:4(8Z,11Z,14Z,17Z)) | 544.3388767 | C28H50NO7P | 7.7036 |
| pos_138 | LysoPC(16:1(9Z)) | 494.3233547 | - | 7.560116667 |
| pos_331 | PC(18:2(9Z,12Z)/18:2(9Z,12Z)) | 782.5676741 | C44H80NO8P | 10.43935 |
| pos_342 | PC(18:0/20:4(8Z,11Z,14Z,17Z)) | 810.599091 | C46H84NO8P | 11.18501667 |
| pos_616 | 1-Linoleoylglycerophosphocholine | 520.3385644 | C27H53NO7P | 7.7749 |
| pos_1283 | PC(16:0/20:4(5Z,8Z,11Z,14Z)) | 804.5499407 | C44H80NO8P | 10.739 |
| pos_3264 | PE(16:0/22:5(7Z,10Z,13Z,16Z,19Z)) | 730.5235003 | C43H76NO8P | 6.349633333 |
| pos_4218 | PC(16:0/22:6(4Z,7Z,10Z,13Z,16Z,19Z)) | 806.5674193 | C46H80NO8P | 10.52356667 |
| pos_5377 | LysoPC(18:0) | 546.3524004 | C26H54NO7P | 8.917983333 |
| pos_5874 | L-Palmitoylcarnitine | 400.3415326 | C23H45NO4 | 7.408483333 |
| pos_7157 | PE(14:0/22:6(4Z,7Z,10Z,13Z,16Z,19Z)) | 718.4873528 | C41H70NO8P | 4.943366667 |
| pos_7176 | PE(14:0/20:4(5Z,8Z,11Z,14Z)) | 676.4758311 | C39H70NO8P | 4.9136 |
| neg_201 | 12(R)-HETE | 319.2265323 | - | 7.478116667 |
| neg_756 | LysoPC(18:3(9Z,12Z,15Z)) | 562.3134118 | C26H48NO7P | 7.307233333 |
| neg_785 | LysoPC(16:1(9Z)/0:0) | 538.3134614 | C24H48NO7P | 7.560283333 |
| neg_999 | PC(18:1(11Z)/18:3(6Z,9Z,12Z)) | 826.5586321 | C44H80NO8P | 10.4341 |
| neg_1035 | PE(15:0/22:1(13Z)) | 804.5749688 | C42H82NO8P | 11.28438333 |
| neg_1159 | PE(15:0/24:1(15Z)) | 832.6063319 | C44H86NO8P | 11.84768333 |
| neg_3725 | PC(20:2(11Z,14Z)/18:1(11Z)) | 856.6065374 | C46H86NO8P | 11.43021667 |
| neg_3799 | PC(16:0/20:3(5Z,8Z,11Z)) | 828.5748635 | C44H82NO8P | 10.92773333 |

Table S2 Differential metabolite KEGG pathway - Synthesis and metabolism of polysaccharide 5 metabolites

| ID | Metabolite | Library ID | M/Z | Formula | Retention time |
| --- | --- | --- | --- | --- | --- |
| pos_3264 | PE(16:0/22:5(7Z,10Z,13Z,16Z,19Z)) | HMDB0008945 | 730.5235003 | C43H76NO8P | 6.349633333 |
| pos_7157 | PE(14:0/22:6(4Z,7Z,10Z,13Z,16Z,19Z)) | HMDB0008847 | 718.4873528 | C41H70NO8P | 4.943366667 |
| pos_7176 | PE(14:0/20:4(5Z,8Z,11Z,14Z)) | HMDB0008838 | 676.4758311 | C39H70NO8P | 4.9136 |
| neg_1035 | PE(15:0/22:1(13Z)) | HMDB0008908 | 804.5749688 | C42H82NO8P | 11.28438333 |
| neg_1159 | PE(15:0/24:1(15Z)) | HMDB0008915 | 832.6063319 | C44H86NO8P | 11.84768333 |

Table S3 Differential metabolite KEGG pathway - tumor correlation 15

| ID | Metabolite | Metab ID | M/Z | Formula | Retention time |
| --- | --- | --- | --- | --- | --- |
| pos_135 | LysoPC(22:6(4Z,7Z,10Z,13Z,16Z,19Z)) | metab_135 | 568.3383944 | C30H50NO7P | 7.589166667 |
| pos_137 | LysoPC(20:4(8Z,11Z,14Z,17Z)) | metab_137 | 544.3388767 | C28H50NO7P | 7.7036 |
| pos_138 | LysoPC(16:1(9Z)) | metab_138 | 494.3233547 | - | 7.560116667 |
| pos_331 | PC(18:2(9Z,12Z)/18:2(9Z,12Z)) | metab_331 | 782.5676741 | C44H80NO8P | 10.43935 |
| pos_342 | PC(18:0/20:4(8Z,11Z,14Z,17Z)) | metab_342 | 810.599091 | C46H84NO8P | 11.18501667 |
| pos_589 | L-Isoleucine | metab_589 | 132.1018405 | C6H13NO2 | 0.734616667 |
| pos_616 | 1-Linoleoylglycerophosphocholine | metab_616 | 520.3385644 | C27H53NO7P | 7.7749 |
| pos_1283 | PC(16:0/20:4(5Z,8Z,11Z,14Z)) | metab_1283 | 804.5499407 | C44H80NO8P | 10.739 |
| pos_4218 | PC(16:0/22:6(4Z,7Z,10Z,13Z,16Z,19Z)) | metab_4218 | 806.5674193 | C46H80NO8P | 10.52356667 |
| pos_5377 | LysoPC(18:0) | metab_5377 | 546.3524004 | C26H54NO7P | 8.917983333 |
| neg_756 | LysoPC(18:3(9Z,12Z,15Z)) | metab_9226 | 562.3134118 | C26H48NO7P | 7.307233333 |
| neg_785 | LysoPC(16:1(9Z)/0:0) | metab_9255 | 538.3134614 | C24H48NO7P | 7.560283333 |
| neg_999 | PC(18:1(11Z)/18:3(6Z,9Z,12Z)) | metab_9469 | 826.5586321 | C44H80NO8P | 10.4341 |
| neg_3725 | PC(20:2(11Z,14Z)/18:1(11Z)) | metab_12195 | 856.6065374 | C46H86NO8P | 11.43021667 |
| neg_3799 | PC(16:0/20:3(5Z,8Z,11Z)) | metab_12269 | 828.5748635 | C44H82NO8P | 10.92773333 |
